# Multi-Scale Structural Assessment of Cellulose Fibres Cement Boards Subjected to High Temperature Treatment

**DOI:** 10.3390/ma12152449

**Published:** 2019-08-01

**Authors:** Tomasz Gorzelańczyk, Michał Pachnicz, Adrian Różański, Krzysztof Schabowicz

**Affiliations:** Faculty of Civil Engineering, Wrocław University of Science and Technology, Wybrzeże Wyspiańskiego 27, 50-370 Wrocław, Poland

**Keywords:** cellulose fibre cement boards, microstructure, nanoindentation, SEM-EDS analysis, temperature

## Abstract

The methodology of multi-scale structural assessment of the different cellulose fibre cement boards subjected to high temperature treatment was proposed. Two specimens were investigated: Board A (air-dry reference specimen) and Board B (exposed to a temperature of 230 °C for 3 h). At macroscale all considered samples were subjected to the three-point bending test. Next, two methodologically different microscopic techniques were used to identify evolution (caused by temperature treatment) of geometrical and mechanical morphology of boards. For that purpose, SEM imaging with EDS analysis and nanoindentation tests were utilized. High temperature was found to have a degrading effect on the fibres contained in the boards. Most of the fibres in the board were burnt-out, or melted into the matrix, leaving cavities and grooves which were visible in all of the tested boards. Nanoindentation tests revealed significant changes of mechanical properties caused by high temperature treatment: “global” decrease of the stiffness (characterized by nanoindentation modulus) and “local” decrease of hardness. The results observed at microscale are in a very good agreement with macroscale behaviour of considered composite. It was shown that it is not sufficient to determine the degree of degradation of fibre-cement boards solely on the basis of bending strength; advanced, microscale laboratory techniques can reveal intrinsic structural changes.

## 1. Introduction

Fibre-cement boards were invented by the Czech engineer Ludwik Hatschek over 100 years ago. They have been used in construction as siding, ceilings, floors, roofs and tile backer boards because they are damp-proof and nonflammable light-weight, strong and durable. Nowadays the cellulose fibre cement boards belong to a special class of fibre-reinforced cementitious composites and they consist of 50–70% of cement while the other components include: mineral fibres (usually cellulose) and fillers (limestone powder, kaolin, etc.). The mechanical properties, durability and microstructure of fibre-cement boards in the various environments are widely described in the literature [1,2]. The final properties of cellulose fibre cement composites depend, aside from the fibre and the matrix components, on the manufacturing process as well as on the internal microstructure.

Chady, Schabowicz et al. [3,4] proposed various non-destructive testing methods for evaluating fibre-cement boards as to the potential occurrence of heterogeneities or defects in them. Tonoli et al. [5] analyzed the effects of natural weathering on microstructure and mineral composition of cementitious roofing tiles reinforced with fique fibre. Savastano et al. [6] tested microstructure and mechanical properties of waste fibre-cement composites. At the same time the X-ray microtopography technique has been developed to study the microstructures for non-destructive characterization of the internal structure of various materials [7,8]. Cnudde et al. [9] were using the micro-CT method to determine the impregnation depth of water repellents and consolidants inside natural building stones. Li-Ping Guo et al. [10] investigated the effects of mineral admixtures on initial defects existing in high-performance concrete microstructures using a high-resolution X-ray micro-CT. Wang et al. [11] used this technique to produce the X-ray tomography images of porous metal fibre sintered sheet with 80% porosity. 3D information about the total porosity and the pore size distribution was obtained with the combination of micro-CT and home-made 3D software [12]. Schabowicz, Ranachowski at al. [13,14] successfully used the micro computed tomography (micro-CT) and SEM in the quality control system of cellulose fibre distribution in cement composites. Ranachowski and Gorzelańczyk et al. [15,16,17] investigate the degradation of the microstructure and mechanical properties of fibre cement board (FCB), which was exposed to environmental hazards, resulting in thermal impact on the microstructure of the board. Visual light and scanning electron microscopy, X-ray micro tomography, flexural strength, and work of flexural test *W_f_* measurements were used.

From the literature review presented above, it is clear that until now the mechanical parameters of the microstructure of cellulose fibres cement boards have not been studied. It is however of primary importance to identify the mechanical morphology at microscale which directly affects the mechanical behavior of material at macroscale, i.e., at the scale of engineering applications. In the case of composite materials with a cement matrix, segmentation and characterization of components’ mechanical properties is impossible with the use of classical macro scale experiments [18]. Therefore, advanced laboratory techniques that allow observation of the mechanical behavior of materials at different scales are used very often nowadays. An example of such technique is a classical nanoindentation test developed by Oliver and Pharr as a method to accurately calculate hardness and elastic modulus from the “load-displacement” curve [19].

The idea of nanoindentation is to determine the mechanical properties of composite components by observing their reaction to the point load followed by continuous unload of its surface. Initially, due to hardware limitations, the nanoindentation technique was mainly used in relatively homogeneous media, or layered materials with known thickness of individual layers [20,21]. Currently, better hardware capabilities have made it possible to use this technique for microheterogeneous materials, exhibiting different mechanical morphology depending on the observation scale [22,23]. Nanoindentation tests were successfully used to identify mechanical morphologies for different types of cementitious materials in the works [24,25,26].

This paper presents a multiscale approach for identification of internal structural changes of cellulose fibres cement boards subjected to high temperature treatment. Two advanced laboratory techniques are used, i.e., SEM imaging with EDS analysis and nanoindentation tests. The former technique provides microphotographs of the applied fibres together with the elemental composition of tested samples. The latter (nanoindentation) reveals the mechanical morphology of the material in terms of hardness and elastic moduli. All tests were carried out on two samples: reference material at air-dried state, and the one subjected to high temperature treatment. An evolution of geometrical and mechanical morphology of investigated boards is observed and discussed.

## 2. Materials and Methods

**Figure 1:**
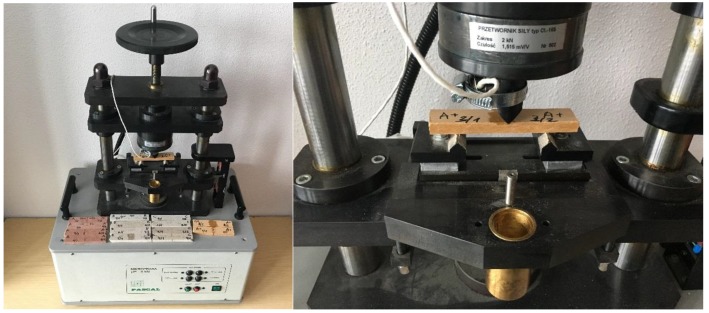
Test stand for bending strength measurements, and fibre-cement board during test.

In this study, two sets of specimens were prepared for examination. These were labeled A and B. The specimens were fabricated by applying the Hatschek forming method. Air-dry reference specimens (not subjected to high temperature treatment) were denoted as A. The specimens, which underwent the high temperature treatment in an electric oven for 3 h at a temperature of 230 °C, were labeled as B. The parameters of the heating procedure were chosen experimentally, after performing some preliminary tests to evoke considerable changes in the microstructure of the investigated materials. The tested specimens were cut from the different fibre cement panels of 8 mm of thickness. Prior to the main research the panels were tested using the standard procedures to assess their performance. Comparisons of tested panels are presented in Table 1 and the set of equipment for bending strength measurements is shown in Figure 1.

**Figure 2:**
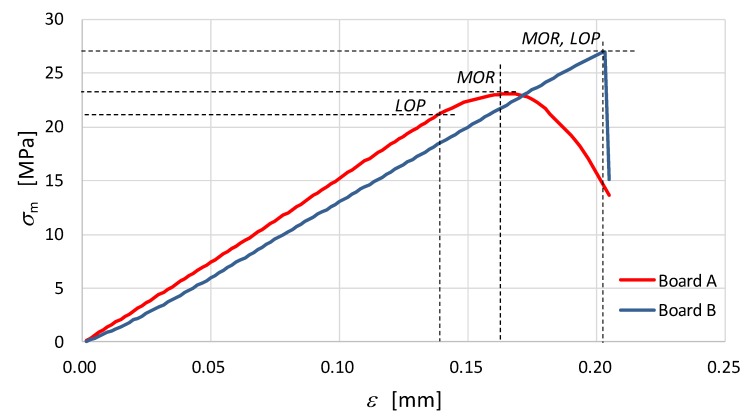
Diagrams of the *σ*-*ε* dependence under bending for fibre-cement boards.

Figure 2 shows an exemplary *σ*-*ε* curve of the tested fibre-cement boards under bending. The trace of flexural strength *σ*, bending strength *MOR*, limit of proportionality (*LOP*) and strain *ε* were analyzed. Bending strength *MOR* was calculated from the standard formula [27]:

(1) $$MOR\;=\;\frac{3Fl_{s}\;}{2b\;e^{2}}$$

where:

*F* is the loading force (N);
*l_s_* is the length of the support span (mm);
*b* is the specimen width (mm); and
*e* is the specimen thickness (mm).

In case of microscale experiments, first, the analysis was performed by SEM imaging with EDS elemental composition examination. For that purpose, the authors have prepared fractured specimens to produce the SEM micrographs applying the Quanta FEG-250 Scanning Electron Microscope (Hillsboro, OR, USA), FEI with an EDS analyser. The precondition was made at 50% of relative humidity and 22 °C to enable for different modes of decomposition of composite microstructure.

**Figure 3:**
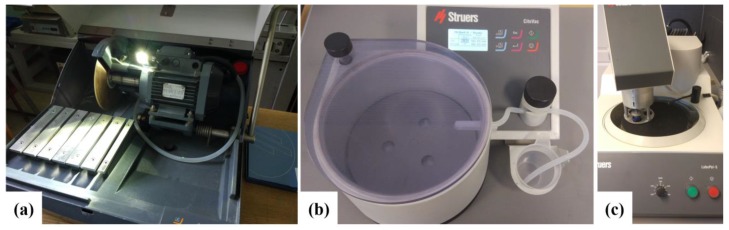
Equipment used for sample preparation: (**a**) Struers Labotom-5, (**b**) Struers CitoVac, (**c**) Struers LaboPol-5.

**Figure 4:**
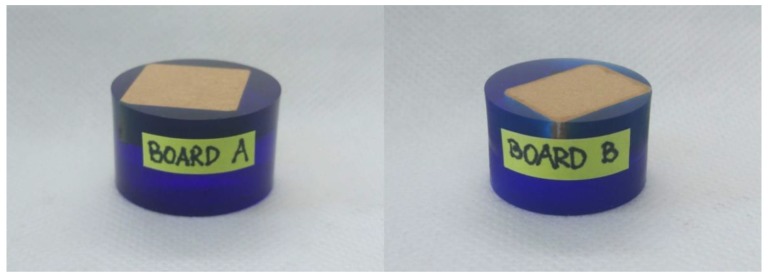
Samples prepared for the indentation.

Next the boards were investigated in terms of nanoindentation approach. It is commonly known that the process of sample preparation for nanoindentation is of primary importance for getting reasonable results. In general, we can assume that the results from nanoindentation can only be as good as the sample used for testing. Hence, the aim of the preparation process was to obtain a satisfactory quality of: parallelism and roughness of surfaces, cleanness of the sample and sample tilt. We followed a common rule-of-thumb contained in the ISO standard for nanoindentation (ISO 14577) as well as requirements, concerning surface roughness criteria for cement paste nanoindentation, provided in [25]. Preparation of the samples for nanoindentation consisted of cutting fibre boards to required dimensions (approximately 1 × 1 cm), mounting specimens in the epoxy resin and thorough grinding and polishing process of the specimen surface. High-speed diamond saw Struers Labotom-5 (Copenhagen, Denmark) (Figure 3a), Struers CitoVac vacuum chamber (Copenhagen, Denmark) (Figure 3b) and Struers LaboPol-5 grinder (Copenhagen, Denmark) (Figure 3c) were used for the sample preparation. The photos of the samples after the preparation procedure are presented in Figure 4.

**Figure 5:**
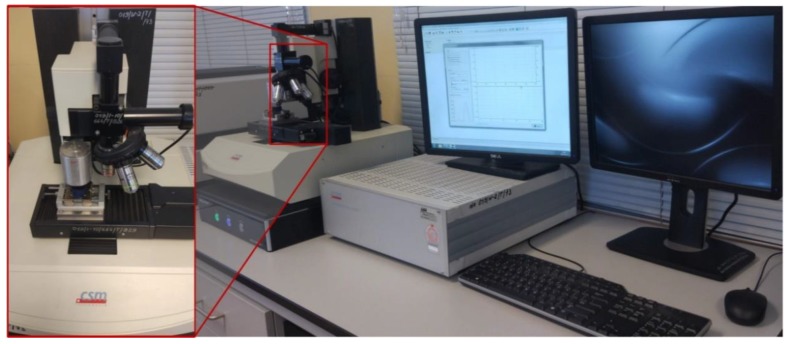
Nanoindentation test stand.

Nanoindentation tests were performed using Nanoindenter CSM TTX-NHT (Neuchatel, Switzerland) (Figure 5) equipped with a diamond Berkovich tip (Poisson’s ratio *ν_i_* = 0.07, elastic modulus *E_i_* = 1000 GPa, *β* = 1.034).

## 3. Results of Multiscale Approach

### 3.1. SEM Analysis

**Figure 6:**
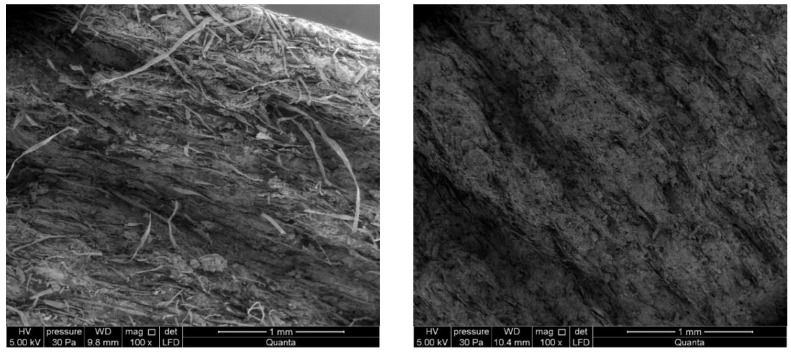

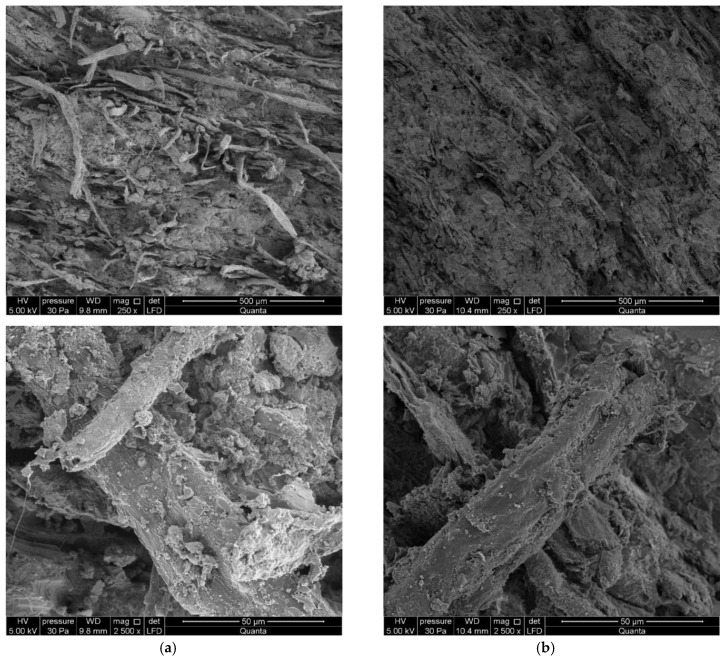
The microscopic observations of the applied fibres, in scanning electron microscope (SEM) with scale bar in sequence 1 mm, 500 µm and 50 µm: (**a**) air-dry condition (reference board), (**b**) board exposure to temperature of 230 °C-3 h.

In this Section the results obtained with SEM imaging are presented. In particular, Figure 6 shows the microphotographs of the applied fibres. Note that left (right) panel of Figure 6 presents the microstructure of reference (subjected to high temperature) sample.

**Figure 7:**
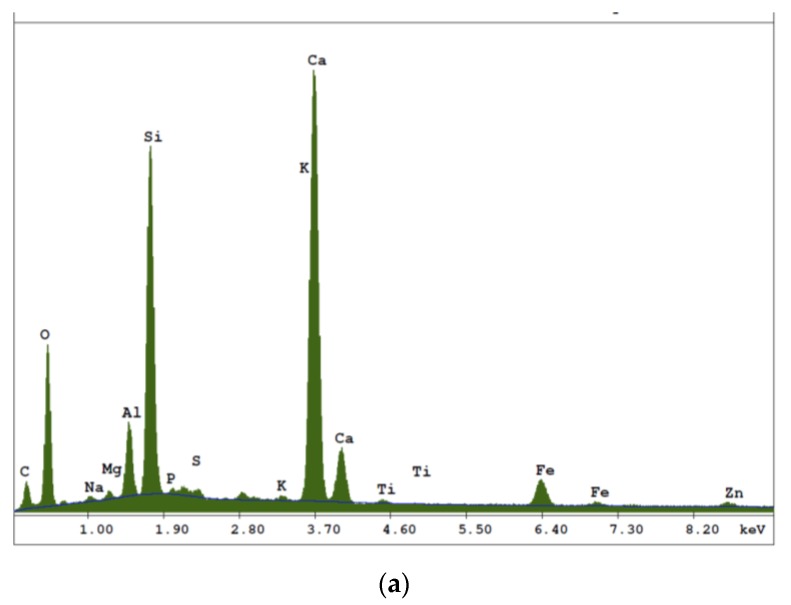

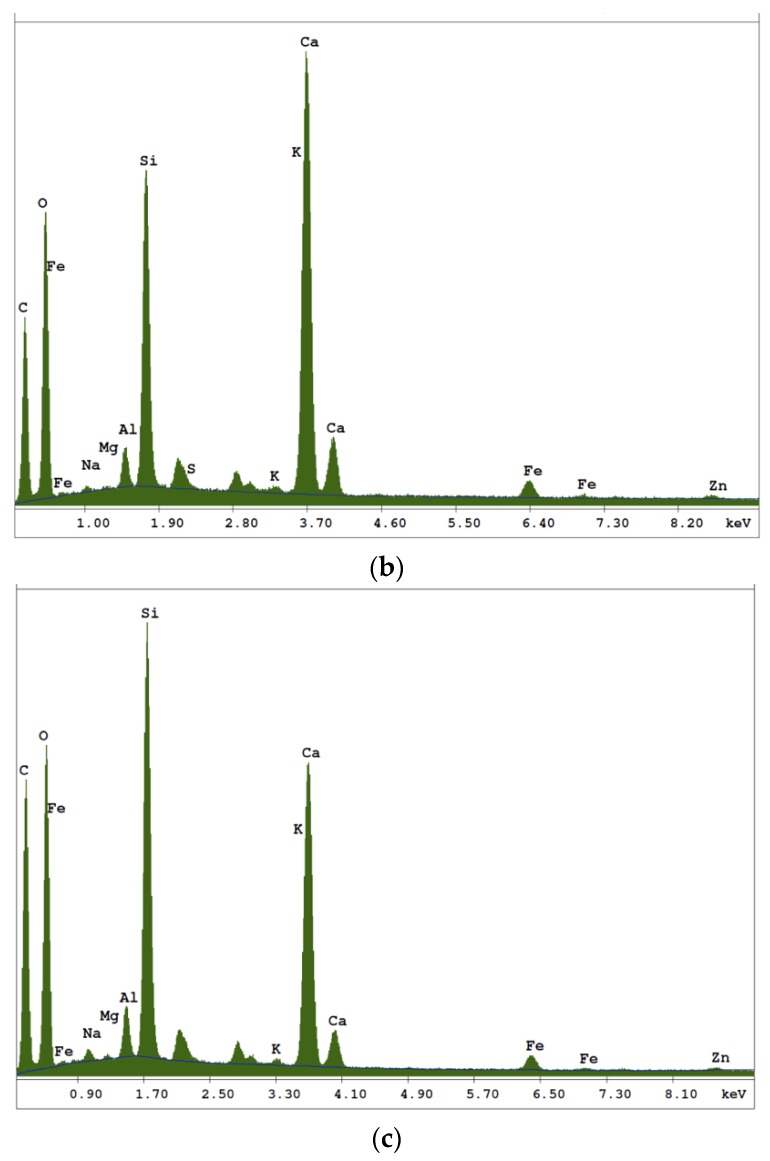
Composition analysis (EDS) of tested boards: (**a**) matrix, (**b**) fibre of air-dry condition (reference board) and (**c**) “fibre” of board exposure to temperature of 230 °C-3 h.

In Figure 7 elemental composition (results of EDS analysis) of tested samples is graphically presented. The investigation was carried out separately for matrix (Figure 7a), fibre of air-dry condition (reference board, Figure 7b) “fibre” of board exposed to temperature of 230 °C for 3 h (Figure 7c).

### 3.2. Nanoindentation

**Figure 8:**
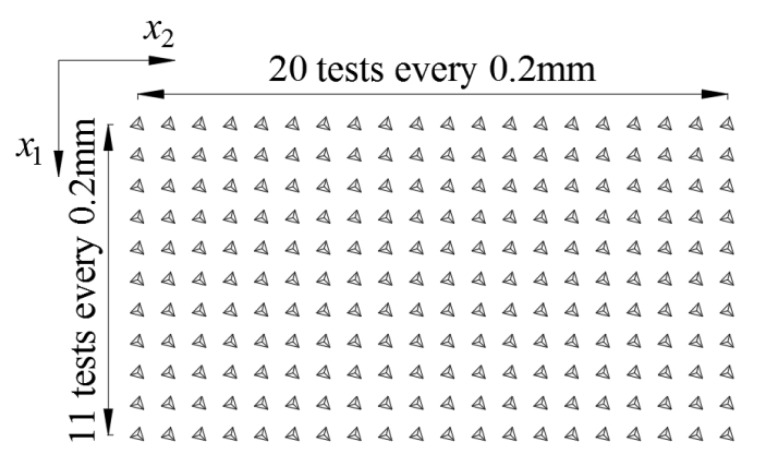
Applied nanoindentation grid.

The assessment of mechanical parameters of the fibre cement boards was carried out using the method formulated for heterogeneous materials with a cement matrix. This technique, the Grid Indentation Technique (GIT), was introduced in [18]. For each specimen a regular grid of 220 tests was applied (Figure 8). The relevant grid parameters, such as the distance between indenter locations, the number of tests and the single test’s maximum load were determined by trial nanoindentation tests in accordance with the recommendations presented in [18].

**Figure 9:**
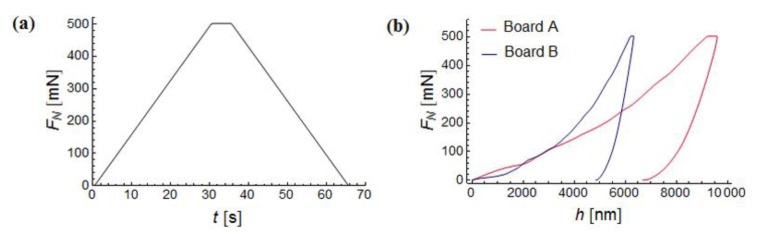
(**a**) Load function used for single nanoindentation, (**b**) and example of *F-h* curve.

In every grid point, a single standard nanoindentation test [19,28,29] was performed with the maximum load of 500 mN. The test runs as follows: continuous increase of force up to a fixed value *F*_max_, then a short period in which the set maximum value of force is maintained, after that it is followed by unloading which is also carried out continuously (Figure 9a). During the test, the relation between the force *F* and the depth of penetration *h* is recorded. An example of *F*-*h* curve obtained for a single test is presented in Figure 9b. For every test two indentation parameters were calculated, namely hardness (*H*_IT_) defined as follows:

(2) $$H_{\mathrm{IT}}=\frac{F_{\max}}{A}$$

and the indentation modulus (*M*_IT_):

(3) $$M_{\mathrm{IT}}=\frac{S}{2\beta }\frac{\sqrt{\pi }}{\sqrt{A}}$$

where, *F*_max_ is the maximum force of indentation, *A* is the projection of the contact area on the surface of the sample. This value is usually defined as a function of the maximum indentation depth *h*_max_ [18] and *S* is the initial slope of the unloading curve according to [18].

**Figure 10:**
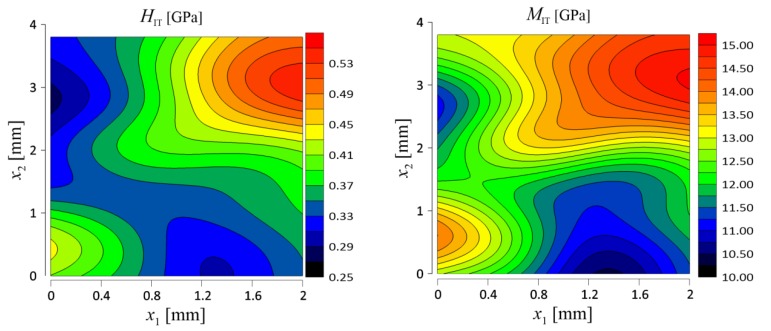
Distribution of hardness and indentation modulus for Board A (reference material).

**Figure 11:**
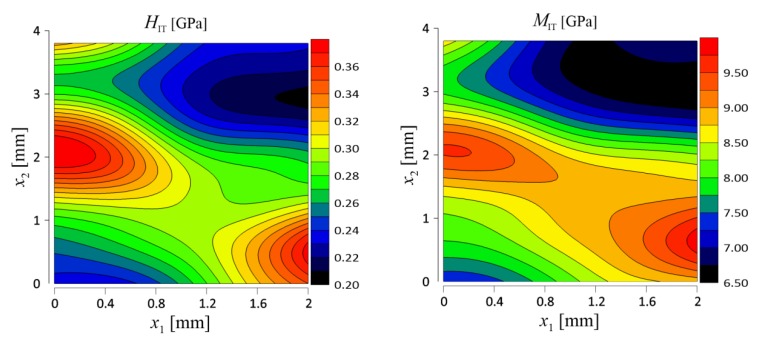
Distribution of hardness and indentation modulus for Board B.

As a consequence of performed tests, distribution of the mechanical parameters on the surface of the samples was evaluated. Contour maps of hardness and indentation modulus distribution are presented in Figure 10 and Figure 11 for Board A and Board B, respectively.

**Figure 12:**
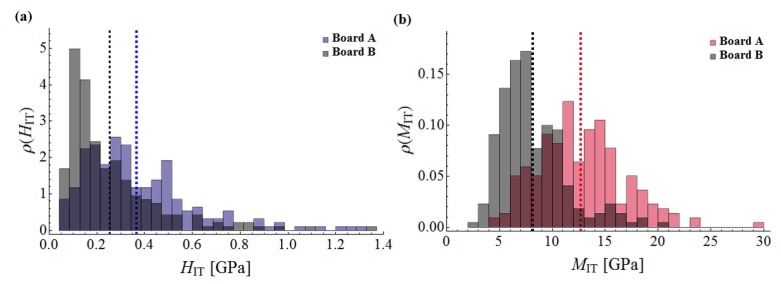
(**a**) Histograms of hardness; (**b**) histograms of indentation modulus.

All obtained values are presented in the form of histograms in Figure 12a,b. The results corresponding to the case of high temperature heating (230 °C-3 h, Board B) are displayed in grey. Red and blue colors are corresponding to the results obtained for reference samples (Board A). In particular, the red color represents the distribution of the indentation modulus, whereas the blue one represents the frequency of hardness values. The vertical dashed lines refer to the mean values averaged over 220 individual results. Furthermore, exact values of statistical measures, i.e., mean values *µ* and standard deviations *σ*, are summarized in Table 2.

**Figure 13:**
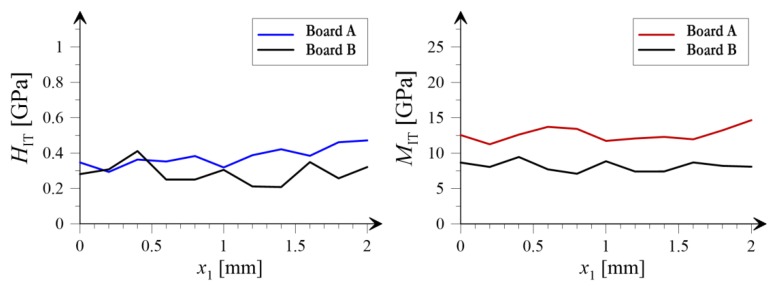
Average values of hardness (*H*_IT_) and indentation modulus (*M*_IT_) in the *x*_2_ direction.

**Figure 14:**
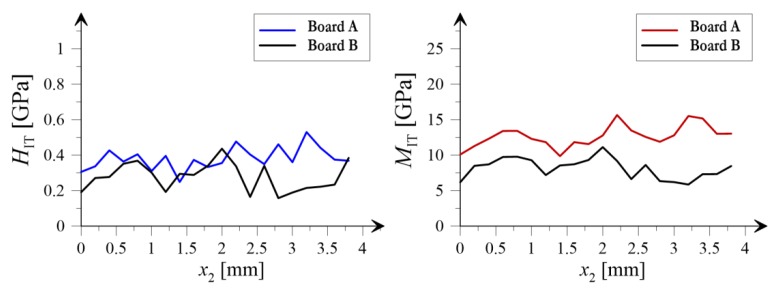
Average values of hardness (*H*_IT_) and indentation modulus (*M*_IT_) in the *x*_1_ direction.

In Figure 13 and Figure 14 the variation of mean values averaged along two independent directions, respectively for *x*_1_ and *x*_2_ (see coordinate system shown in Figure 8), is presented. The black color corresponds to the board subjected to heating and blue (hardness) and red (indentation modulus) colors are representing the results obtained for the reference sample.

## 4. Discussion of Results

The results presented in Table 1 and Figure 2 show that, under high temperature, the bending strength *MOR* increases. The increase (in average sense) from 23.54 to 26.86 MPa is observed. For fibre-cement Board B, it was also observed that under the influence of a temperature of 230 °C, the structure of the Board Becomes more brittle; after peak value of stress, a sudden drop of strength is observed (Figure 2). An analysis of the classical macroscale results showed that for fibre-cement Board B the extent of the nonlinear increase in bending stress is reduced until the bending strength *MOR* comes to be level with the proportionality limit *LOP*. In the case of the reference board, the *MOR* and *LOP* values were clearly separated. It should be noted that the reference samples were under an air-dry condition and their bulk moisture content amounted to 6–8%.

Based on the macroscale results (bending strength) one can draw the conclusion that after exposure to the temperature (230 °C for 3 h) there is no degradation of the board, and even the strengthening effect is obtained. Nevertheless, advanced microscale laboratory techniques have revealed damaging and irreversible structural changes in both geometrical and mechanical morphology of microstructure.

An analysis of the images obtained from the scanning electron microscope and the EDS analyzer shows that the fibre cement Board A has a compact microstructure (Figure 6a). Microscopic examinations revealed a fine-pore structure, with pores of up to 50 µm in size. Cavities and grooves, up to 500 µm wide, were visible in the fracture areas where the fibres had been pulled out. Cellulose fibres and PVA fibres, are clearly visible in the images. Various forms of hydrated calcium silicates of the C-S-H type occur. Both an “amorphous” phase and a phase built of strongly-adhering particles predominate. An analysis of the fibre composition showed that fibre elements and some cement elements were present. An analysis of the chemical composition of the matrix showed elements that are typical of cement (Figure 7). The surface of the fibres was covered with a thin layer of cement paste and hydration products. The fact that there are very few areas with a space between the fibres and the cement paste, indicates that the fibre-cement bond is strong.

A microscopic analysis of the fibre cement Board B, which was exposed to a temperature of 230 °C for 3 h, shows a clear change in the colour of the samples (Figure 6b). Most of the fibres in the board were found to be burnt-out, or melted into the matrix, leaving cavities and grooves which were visible in all of the tested boards. The structure of the few remaining fibres was strongly degraded. An examination of the cement particles on the fracture surface revealed burning-out of their structure. The structure of the matrix was found to be more granular, showing many delaminations (Figure 6b). Numerous caverns and grooves left by the pulled-out fibres, as well as the pulled-out cement particles, were observed.

Noticeable changes of mechanical morphology of the analyzed boards due to the influence of high temperature is observed within the nanoindentation approach. The average value of the indentation modulus, being the measure of the elastic response of the material, after the exposure to the temperature of 230 °C for 3 hours, significantly decreased (see Figure 12b). The histogram for the Board B (grey colour) concentrates around lower values of modulus compared with the histogram for Board A. In addition, according to Table 2 the average value of the indentation modulus, after heating at the temperature of 230 °C, decreased from 12.686 GPa to 8.147 GPa which equals to an almost 50% drop in the elastic stiffness of examined material. Furthermore, as shown in the right panels of Figure 13 and Figure 14, the mean value of indentation modulus is significantly smaller for Board B compared to the values obtained for Board A in the entire range of *x*_1_ and *x*_2_ values. These observations are in a very good agreement with SEM analysis. A decrease of elastic response of material’s microstructure, its stiffness in fact, is mostly due to the changes in the microstructure geometry observed in SEM. The presence of cavities and grooves in Board B can be a direct cause of decrease in *M*_IT_ values. It is worth noticing that the changes of geometrical (SEM analysis) and mechanical (nanoindentation) morphology of Board B is a direct cause of the macroscale behavior; by observing Figure 2, one can simply notice that the stiffness of Board B is reduced compared to the one evaluated for Board A (the slope of *σ*-*ε* curve decreased after exposure to the temperature); and it was observed in case of all boards under bending.

Slightly different conclusions can be drawn for the hardness of the material. In general, the average hardness value, as a result of exposure to the temperature 230 °C, decreased, from 0.382 GPa for the Board A, to 0.285 GPa for the Board B (see Figure 12a and Table 2). On the other hand, observing the left panels of Figure 13 and Figure 14 one can notice that “locally”, i.e., for given ranges of *x*_1_ or *x*_2_ values, the decrease of *H*_IT_ is not observed. As shown in [30,31] it is the hardness *H*_IT_ which is the parameter determining the strength of the microstructure. This is due to fact that the strength is proportional to the hardness of individual microstructure components; the higher the hardness, the higher the strength of microstructure. Therefore the phenomenon observed e.g., in the left panels of Figure 13 (in the range of *x*_1_ from 0 to 0.5 mm) and Figure 14 (in the range of *x*_2_ from 0.5 to 2.0 mm), where no decrease in *H*_IT_ is observed, can justify the macroscale bending strength results. As shown in Table 1, the bending strength of Board B is slightly higher than the one evaluated for Board A; this is in the average sense since the values in Table 2 represent mean values. However, during individual bending tests we also observed the cases when the strength was not increased or even slightly decreased. This is the effect of the fact that “locally” hardness does not change under the influence of temperature, and hence, this can cause such macroscopic behavior of the boards.

It should also be mentioned that the standard deviations for both the indentation modulus and the hardness of the specimen subjected to temperature 230 °C slightly decreased. However, as mentioned above, the mean values of both properties decreased in a more evident manner, and hence the mechanical morphology of Board B seems to have more heterogeneous nature. This is clearly revealed by plots shown in Figure 13 and Figure 14 as well as by c.o.v. (coefficient of variation) values summarized in Table 2. For Board B, the coefficient of variation of both hardness and indentation modulus increased.

## 5. Final Conclusions

Two methodologically different microscopic techniques were used to identify the evolution of geometrical and mechanical morphology of boards. The methodology of multi-scale structural assessment of the different cellulose fibre cement boards subjected to high temperature treatment was presented. For that purpose, two specimens were investigated: Board A (air-dry reference specimen) and Board B (exposed to a temperature of 230 °C for 3 h).

SEM examinations were carried out to get a better insight into the changes taking place in the structure of the tested boards. Significant changes take place in the structure of the boards, especially after the high temperature treatment in an electric oven for 3 h at a temperature of 230 °C. Most of the fibres in the board were burnt-out, or melted into the matrix, leaving cavities and grooves which were visible in all of the tested boards. The structure of the few remaining fibres was strongly degraded, as confirmed by the nanoindentation tests. Nanoindentation tests revealed significant changes of mechanical properties caused by high temperature treatment: “global” decrease of the stiffness (characterized by nanoindentation modulus) and “local” decrease of hardness. The results observed at microscale are in a very good agreement with macroscale behaviour of considered composite.

In the authors’ opinion, the above findings are important for building practice because it was clearly shown that it is not sufficient to determine the degree of degradation of fibre-cement boards solely on the basis of bending strength. Based only on the macroscale results (bending strength) one can draw the conclusion that after exposure to the temperature (230 °C for 3 h) there is no degradation of the board. Moreover, in the average sense, some strengthening effect can also be observed. Microscale laboratory techniques adapted in this work, however, reveal damaging and irreversible microstructural changes of boards caused by the high temperature treatment.

It should be also noticed that the presented results are preliminary and starting a research cycle. Based on them, changes in mechanical parameters, especially of fibres in the fibre cement board, after exposed to a temperature of 230 °C for 3 h have been demonstrated. Currently, studies are being carried out to show the impact of high-temperature on the fibre cement board, but in a much shorter time. The authors hope to publish promising results soon.

## Figures and Tables

**Table 1:** Characteristic of the tested panels.

Symbol of the Board	A	B
Type of the board	fibre-cement, exterior	fibre-cement, exterior, after 3 h of burning (230 °C)
Thickness of board [mm]	8	8
Bending strength [MPa]	23.54 *	26.86 *
Density [kg/m^3^]	1600	1500
Photo of the board	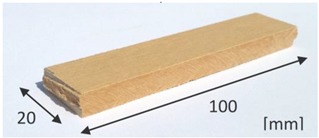	

* mean values calculated on the basis of ten independent measurements.

**Table 2:** Summary of nanoindentation results.

Parameter		Specimen before High Temperature Treatment (Board A)	Specimen after High Temperature Treatment (Board B)
Mean value of hardness	$\mu_{}^{H_{\mathrm{IT}}}$ [GPa]	0.382	0.285
Standard deviation of hardness	$\sigma_{}^{H_{\mathrm{IT}}}$ [GPa]	0.226	0.201
Coefficient of variation * of hardness	$c.o.v^{H_{IT}}$	0.592	0.705
Mean value of indentation modulus	$\mu_{}^{M_{\mathrm{IT}}}$ [GPa]	12.686	8.147
Standard deviation of indentation modulus	$\sigma_{}^{M_{\mathrm{IT}}}$ [GPa]	4.093	3.138
Coefficient of variation * of indentation modulus	$c.o.v^{M_{IT}}$	0.318	0.385

* Coefficient of variation is defined as the ratio of the standard deviation to the mean value.
